# Uptake of prevention of mother-to-child-transmission using Option B+ in northern rural Malawi: a retrospective cohort study

**DOI:** 10.1136/sextrans-2013-051336

**Published:** 2014-04-08

**Authors:** Alison J Price, Michael Kayange, Basia Zaba, Frank M Chimbwandira, Andreas Jahn, Zengani Chirwa, Aisha NZ Dasgupta, Cynthia Katundu, Jacqueline L Saul, Judith R Glynn, Olivier Koole, Amelia C Crampin

**Affiliations:** 1 London School of Hygiene and Tropical Medicine, London, UK; 2 Malawi Ministry of Health, Lilongwe, Malawi; 3 Karonga Prevention Study, Chilumba, Malawi

**Keywords:** ANTENATAL HIV, PREVENTION, CHILDREN

## Abstract

To identify points of dropout on the pathway from offering HIV testing to maintenance on antiretroviral therapy (ART), following the introduction of the Option B+ policy for pregnant women in Malawi (lifelong ART for HIV-positive mothers and 6 weeks nevirapine for the infants), a retrospective cohort study within a demographic surveillance system in northern Malawi. Women living in the demographic surveillance system who initiated antenatal care (ANC) between July 2011 (date of policy change) and January 2013, were eligible for inclusion. Women who consented were interviewed at home about their health facility attendance and care since pregnancy, including antenatal clinic (ANC) visits, delivery and postpartum care. Women's reports, patient-held health records and clinic health records were manually linked to ascertain service use. Among 395 women, 86% had tested for HIV before the pregnancy, 90% tested or re-tested at the ANC visit, and <1% had never tested. Among 53 mothers known to be HIV-positive before attending ANC, 15 (28%) were already on ART prior to pregnancy. Ten women tested HIV-positive for the first time during pregnancy. Of the 47 HIV-positive mothers not already on ART, 26/47 (55%) started treatment during pregnancy. All but five women who started ART were still on treatment at the time of study interview. HIV testing was almost universal and most women who initiated ART were retained in care. However, nearly half of eligible pregnant women not on ART at the start of ANC had not taken up the invitation to initiate (lifelong) ART by the time of delivery, leaving their infants potentially HIV-exposed.

## Introduction

Combination antiretroviral therapy (ART) is highly effective for prevention of mother-to-child transmission (PMTCT) of HIV, yet globally an estimated 390 000 children were infected during pregnancy, delivery or breastfeeding in 2011, 90% of whom were in sub-Saharan Africa.^1^ In July 2011, the Malawi Ministry of Health implemented ‘Option B+’ nationally, a policy to initiate ART for life, regardless of CD4 count, for all HIV-positive pregnant or breastfeeding women, with decentralised provider-initiated HIV testing and counselling, and single tablet regimen, triple therapy ART (in the form of tenofovir-lamivudine-efavirenz), delivered at antenatal clinics (ANC). The baby should receive 6 weeks of nevirapine, PCR-based HIV screening at 6 weeks, additional rapid testing at 12 months and 24 months and ART if HIV-positive. Option B+ replaced a modified ‘Option A’ regimen of daily zidovudine during pregnancy and combination lamivudine-nevirapine at delivery for the mother (or in case of home delivery, single dose nevirapine to take home) and 1–4 weeks of daily zidovudine syrup for the infant, depending on the mother's treatment schedule.

This public health approach to PMTCT aims to provide a simplified and standardised regimen^2^ in a country with limited access to CD4 counts,^3^ high fertility (total fertility rate=5.7), and short birth intervals (median=3 years) and where breastfeeding is currently the only feasible option to prevent infant malnutrition.^4^ However, in an overburdened health system relying predominantly on patient-held records (health passport, ART identity card) and clinic registers, and where women are known to change facilities for ANC services and delivery, sharing information between facilities and services to ensure that women and infants who need PMTCT are identified, initiated and retained in care, is a challenge.

We evaluated PMTCT service delivery and uptake in a rural population in the first 18 months (July 2011–January 2013) following policy change, by collecting information retrospectively from women who had given birth and cross-referencing with clinic registers, to established potential points in the system of care where HIV-infected women may fail to be identified or are lost to care services, resulting in more rapid deterioration of the mother's health and potential mother-to-child HIV transmission.

## Methods

The Karonga Prevention Study demographic surveillance system (DSS) in northern Malawi covers an area of 135 km^2^ and a population of 35 000, with linkage to local clinical services.^5^ Four annual HIV serosurveys (2007–2011), with 70% participation and 98% requesting their results, show HIV prevalence is 9% in adult women.^6^ Trained local key informants (n=280) provide notification of all births (including stillbirths) in the DSS on a monthly basis, and the date and location of each birth and the parent's details are recorded at a home visit from a study interviewer. Each key informant covers a ‘cluster’ of about 25 households. Any births missed by an informant are captured during the re-census. Five health facilities provide antenatal services with integrated ART care and four provide routine deliveries (obstetric complications during labour, delivery and the postnatal period are referred to the district hospital, 70 km north of the DSS). Pregnant women are recommended to attend at least four ANC visits, starting before week 17 of pregnancy, and to deliver in a health facility, using a skilled attendant.^4^

A retrospective cohort study was conducted among women resident in the DSS who gave birth (either a live or stillbirth) after the PMTCT policy changed (1 July 2011). All HIV-positive and HIV-unknown women were included. A random sample of HIV-negative women were also included to ascertain whether they were offered HIV counselling and testing (HCT), to avoid inadvertent disclosure of HIV status, and to identify any seroconverters. Mothers who had moved within the DSS area since the registration of a birth were sought at the new location. HCT-trained study interviewers, who did not know the women's HIV status, visited women at home to ask about HIV testing and PMTCT service uptake along the continuum of care (ANC, delivery and postpartum services). Written consent was sought for the interview, for HIV testing if indicated, and for subsequent review of health records at the clinic. Rapid testing was offered to all women unless they reported that they were HIV-positive. All participants were precounselled and those who consented to rapid HIV testing were given post-test counselling. All HIV-positive women not already on ART who were still breastfeeding were interviewed about their reasons for not starting treatment (if previously tested positive) and informed about ART services in the district and given a referral letter.

All women who were HIV-positive (by report or rapid test), or HIV-unknown (no test or last negative test on database prior to 2011), were interviewed about breastfeeding and use of HIV care services. HIV-positive and HIV-unknown mothers who did not have a PCR test recorded in the child's health passport were offered PCR HIV testing of their infant(s), with precounselling. Mothers of babies potentially at risk of mother to child transmission (MTCT) (unknown HIV status, or HIV-positive and no ART) were advised to seek care at a government facility.

The patient-held record and self-report of health centre attendance and service use was checked against clinic registers. If records were not identified at the facility reported, they were sought at all other clinics in the DSS. Identification used the name, age, facility and date of attendance and where available, HCT, ART and delivery identification numbers (recorded in the patient and clinic records by health staff). Authorisation to search the registers was obtained from the district health office and conducted at the convenience of each facility. Ethics approval was obtained from the Malawi National Health Sciences Research Committee and the ethics committee of the London School of Hygiene and Tropical Medicine.

Data were analysed using Stata V.12 software (Stata Corp, College Station, Texas, USA). Data from the HIV serosurveys, self-report, patient-held and clinic records were combined to assess services received, and points of dropout in PMTCT care.

## Results

Among women resident in the DSS who gave birth after 1 July 2011 and before 31 January 2013, 568 were selected to participate. Of these, 35 (6.2%) had left the DSS, 45 (7.9%) could not be found and one (0.2%) had died. Of the 487 women found, 483 (99.2%) consented to interview and review of clinic records. Eighty-eight women had first attended ANC before the policy change so were excluded, leaving 395 women who initiated ANC or did not attend ANC during their pregnancy but delivered after policy change: 53 with a prior HIV-positive test result and 112 with a recent HIV-negative test result on the study database and 230 whose HIV status could not be ascertained from study records (either never tested during the serosurvey or last negative test prior to 2011).

Characteristics of these 395 women are shown in table 1. The mean age was 25 years and 61% had not completed primary education. Patient-held records were available for 80% (316/395); ANC register entries were found for 88% of those who reported at least one ANC attendance in the DSS (338/386; two never attended and seven attended outside the DSS); and delivery register entries were found for 80% of those who reported a hospital birth in the DSS (261/326; 37 delivered at a facility outside the DSS and 32 delivered at home or with a traditional birth attendant). Most women commenced ANC during the second trimester (61%) and attended at least two ANC visits (94%), with some facility switching (9%). Only 47 women (12%) were accompanied to ANC by their husband, and four by other relatives. Almost all women (92%) reported that they delivered at a government health facility (inside or outside the DSS) and 85% used a maternity facility at the same location as their ANC (310/363; data not shown). A third (36%) of women lived >5 km from the nearest health facility.

**Table 1:** Characteristics of 395 women in prevention of mother-to-child-transmission (PMTCT) study by HIV and ART status*

	HIV-positive on ART prior to ANC†	HIV-positive started ART during ANC	HIV-positive not on ART during pregnancy or delivery‡	HIV-negative women§	HIV-unknown¶	Total	Per cent
Total numbers	16	26	21	313	19	395	100
Number of ANC visits							
0	0	0	0	0	2	2	0.5
1	0	1	1	19	1	22	5.6
2	3	8	6	73	1	91	23.0
3	4	5	10	106	8	133	33.7
4+ visits	9	12	4	115	7	147	37.2
Age at delivery (years)							
15–19	0	1	2	99	2	104	26.3
20–24	1	4	2	97	6	110	27.6
25–29	4	6	8	61	6	85	21.5
30+ years	11	15	9	56	5	96	24.3
Highest attained education level							
None/Primary 1–5	2	2	0	20	3	27	6.8
Primary 6–7	7	17	15	164	9	212	53.7
Primary 8	7	6	5	84	3	105	26.6
Secondary 1–3	0	1	1	28	4	34	8.6
Unknown	0	0	0	17	0	17	4.3
First presented at ANC**							
First trimester	0	0	2	18	0	20	5.1
Second trimester	12	17	10	189	13	241	61.3
Third trimester	4	9	9	106	4	132	33.6
Attended ANC with guardian at least once††							
Husband	0	2	1	40	4	47	12.0
Mother/sister	0	0	0	4	0	4	1.0
No guardian (all visits)	16	24	20	269	13	342	87.0
ANC facility							
No switch	15	25	21	281	16	358	91.1
Switch	1	1	0	32	1	35	8.9
Delivered at a health centre††							
Yes	15	23	18	259	16	363	91.9
No	1	3	3	23	2	32	8.1
Offered ART in ANC‡‡							
Offered ART	NA	12	3	NA	NA	15	31.9
Referred to ART clinic	NA	12	9	NA	NA	21	44.7
Not offered or referred	NA	1	2	NA	NA	3	6.4
Unknown§§	NA	1	7	NA	NA	8	17.0
Distance to health centre¶¶ (km)							
<1	2	3	0	26	5	36	9.1
1–3	3	7	5	92	5	112	28.4
3–5	4	7	7	84	2	104	26.3
5+	7	9	9	111	7	143	36.2
Visited Health Centre for PCR***							
Infant age 6–8 weeks	2	2	0	NA	NA	4	6.4
Infant age 9+ weeks	2	0	4	NA	NA	6	9.5
Not done	12	22	12	NA	NA	46	73.0
Unknown	0	2	5	NA	NA	7	11.1
Six weeks oral Nevirapine for child***							
Yes	16	20	8	NA	NA	44	69.9
No	0	5	8	NA	NA	13	20.6
Unknown	0	1	5	NA	NA	6	9.5
Child breastfed							
Yes	16	24	16	NA	NA	56	98.8
No	0	1	0	NA	NA	1	1.7

*HIV status determined by information held in the demographic surveillance site (DSS) HIV database (annual HIV serosurveys 2007–2011), clinic records and a woman's self-report.

†One woman started antiretroviral treatment (ART) while pregnant but before antenatal clinic (ANC).

‡Does not include two women who received a single dose of nevirapine at delivery and one who started ART 6 weeks after delivery.

§Random sample of HIV-negative women in DSS who attended ANC after 1 July 2011.

¶HIV-unknown if no DSS test or clinic record and woman reported no HIV counselling and testing (HCT) during ANC or delivery. Includes two women who did not attend ANC or deliver at a health facility.

**Excludes two women who did not attend ANC.

††Delivered at a health facility inside or outside the DSS.

‡‡Eight women with a positive test on the DSS database concealed their HIV-positive status during ANC and the study interview. Six reported a negative test result in ANC and two reported HCT refusal.

§§An ART start date was found for one of these women.

¶¶Calculated as distance (km) to nearest health centre. HCT, ANC, maternity and ART services available at each centre.

***Information on PCR testing and baby nevirapine was not available for six women who reported a negative status during the study.

In total, 86% reported or were documented to have ever tested for HIV before their most recent pregnancy, 90% tested or retested at ANC or HCT clinic (on the day of an ANC visit), and <1% never tested (data not shown). Of newly diagnosed HIV-positive mothers 80% had previously tested and of women who did not retest during ANC, 9% reported that testing and counselling was not offered by government health staff.

**Figure 1:**
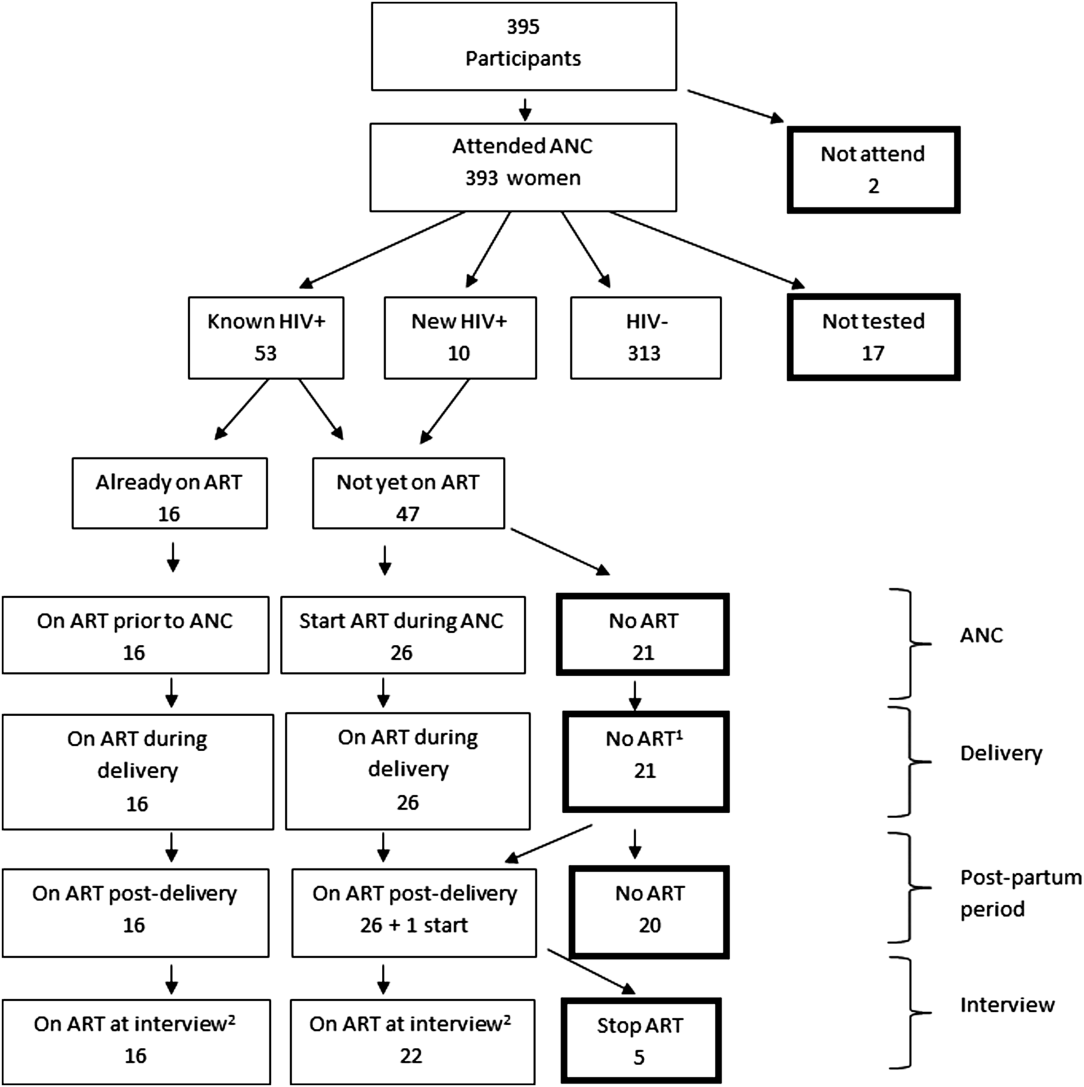
HIV testing and ART uptake and usage among women in antenatal clinic. Boxes with a thick black border highlight the numbers not following the recommended testing and treatment. ^1^Two women received nevirapine at delivery (from residual clinic stock). ^2^ Mean duration of antiretroviral treatment post-delivery was 4.3 months. ANC, Antenatal clinic; ART, antiretroviral therapy; PMTCT, prevention of mother-to-child-transmission.

ART uptake is summarised in figure 1. Of 53 mothers identified via serosurvey or self-report as HIV-positive prior to ANC, 16 (30%) were already on ART before pregnancy. Ten additional women tested HIV-positive for the first time during pregnancy. Among HIV-positive mothers not already on ART, 26/47 (55%) started treatment during pregnancy; almost half (21/47: 45%; table 1) reported they had been referred to a separate ART clinic and eight of these referrals occurred 6 months or more after policy implementation. Eight women known to be HIV-positive from prior study testing concealed their HIV-positive status during ANC by refusing to test and by reporting a negative status during the study interview. However one of these women was found to have started ART during pregnancy. Of 21 HIV-positive women who did not start ART, 14 revealed their HIV-positive status during interview and were asked about ART referral: three reported being offered ART in ANC, nine reported referral to an ART clinic (of whom only two attended and one was assessed but refused treatment; data not shown), and two claimed they had not received advice regarding ART. All of these women reported that ART was not offered at delivery, although four were referred, but they all refused treatment. For 15 of these 21 women there was no record of their HIV-positive status in the ANC register. Reasons reported for not starting ART included fear of disclosure (to husband (8) to others (2)), no trust in ART (10), fear of side effects (6), transport costs (1), preference for herbal treatment (4), inadequate referral from health staff (4) and the belief that ART was not yet needed, due to good health (15). Women could report more than one reason.

Of 63 HIV-positive women, 42/63 (67%) received ART while pregnant and during delivery and among the remaining 21, two received nevirapine during labour and one started ART 6 weeks after delivery. All but one of the at-risk babies were breastfed; at least 20% of these babies did not receive nevirapine syrup and at least 80% of mothers did not return for a PCR test within 6 weeks of delivery (table 1).

Overall, 88% of women (38/43) who had started ART, including all of those already on ART prior to pregnancy, were identified from self-report and clinic records as on treatment at the time of interview (mean 4.3 months (SD 2.3) postdelivery). Of those women who started ART during ANC, 81% (21/26) were retained in care at 6 months.

## Discussion

While these findings should be interpreted with caution due to the small numbers, in the first 18 months of policy change HIV testing was almost universal and all women who had initiated ART prior to pregnancy, and most who had started during ANC, were retained in care. At first ANC attendance 30% of HIV-positive pregnant women were already on ART and this proportion is likely to increase as Option B+ is rolled out. All the children of those women received appropriate treatment at delivery. However 43% of HIV-positive pregnant women not already on ART did not start ART during pregnancy or delivery, and at least 20% of HIV exposed babies did not receive nevirapine.

The high level of HIV testing uptake in ANC is similar to that observed in a recent meta-analysis of opt-out testing strategies in sub-Saharan Africa.^7^ In our population failure to test was largely due to refusal rather than programmatic issues such as staff shortage or unavailability of test kits, as observed elsewhere.^8^ In other populations enhanced HIV testing uptake has been observed among women who attend ANC with their partners.^9^ The effect of male participation on PMTCT uptake in sub-Saharan Africa is not well understood and may vary.^10^ ^11^ In our study, HIV testing uptake was high, and the number who attended with a husband was too small to assess any differences.

The majority of women, including those who did not start ART, attended ANC on three or more occasions, consistent with national surveillance data,^4^ thereby providing several opportunities for ART initiation. However, failure to record positive HIV results in the ANC register—as observed for 71% of those who did not start ART—may contribute to missed opportunities for counselling and initiation of PMTCT at subsequent ANC visits.

While Option B+ aims to deliver ART within ANC services, 45% of eligible women reported that they were referred to separate ART services. Almost half of these referrals occurred more than 6 months after policy change, which suggests challenges associated with provision of the integrated service in the initial stages of the policy. Higher levels of attrition have been observed elsewhere when ART services are provided in separate locations or on different days from ANC, particularly among those living far from the clinic.^12–15^ Higher early attrition has also been observed when women initiate ART to prevent MTCT compared with those who initiate for their own health and among those who initiate at the time of a HIV-positive diagnosis,^16^ which suggests greater need for education (maternal health benefits) and support services to address issues of stigma and disclosure.

In our rural setting nearly 80% of women who initiated ART during the ANC period were retained on treatment 6 months after starting. Lower retention (70%) has been observed in urban and larger health facilities in Malawi.^16^ Retention on ART depends on good referral systems and linkage between facilities. While dropout was low within the time frame of this study, potential barriers to continuation in care may include transportation costs, distance to clinic and human resource constraints resulting in excessive waiting times and scheduling difficulties.^17^ ^18^

Strengths of the study include regular reporting of births by village informants^19^ and continuous demographic surveillance that provide comprehensive birth data for the population. The recent HIV serosurveys (2007–2011) with high levels of female participation and linkage to ART clinic data means that it was possible to identify, with minimal misclassification, almost all HIV-positive and HIV-unknown women in this population who initiated ANC since policy change, and who were known to be or potentially HIV-positive before their most recent birth. Limitations of the study include the small number of HIV-positive women and lack of data on HIV-positive women who experienced an early termination (<7 months) of a pregnancy.

Option B+ is considered a cost-effective strategy for ensuring universal access to ART for PMTCT in Malawi^20^ and has led to substantial increases in women initiating ART during pregnancy.^3^ However, our data suggest that more support is required to facilitate integration of PMTCT services in ANC and to address the reluctance of some healthy women to test or reveal their HIV-positive status in ANC and/or to initiate ART during pregnancy. Studies are needed to explore these barriers and to evaluate the impact of different healthcare delivery strategies, including patient education and support services for women and their families, on uptake and retention. If refusal remains high, alternative interventions will also need to be offered to prevent MTCT.

**Key messages**

- Implementation of the Option B+ policy may be compromised by reluctance of some women to reveal their HIV-positive status in ANC and/or to initiate antiretroviral therapy during pregnancy.
- More support is required to facilitate integration of prevention of mother-to-child-transmission services in ANC.
- Barriers to uptake of Option B+ need to be explored and addressed with targeted health education and support services.
- If refusals remain high, alternative interventions may need to be offered.
